# The role of zebrafish (*Danio rerio*) in dissecting the genetics and neural circuits of executive function

**DOI:** 10.3389/fncir.2013.00063

**Published:** 2013-04-08

**Authors:** Matthew O. Parker, Alistair J. Brock, Robert T. Walton, Caroline H. Brennan

**Affiliations:** School of Biological and Chemical Sciences, Queen Mary University of LondonLondon, UK

**Keywords:** zebrafish, attention, impulsivity, behavioral flexibility, psychiatric disorder, neural circuits

## Abstract

Zebrafish have great potential to contribute to our understanding of behavioral genetics and thus to contribute to our understanding of the etiology of psychiatric disease. However, progress is dependent upon the rate at which behavioral assays addressing complex behavioral phenotypes are designed, reported and validated. Here we critically review existing behavioral assays with particular focus on the use of adult zebrafish to explore executive processes and phenotypes associated with human psychiatric disease. We outline the case for using zebrafish as models to study impulse control and attention, discussing the validity of applying extant rodent assays to zebrafish and evidence for the conservation of relevant neural circuits.

## INTRODUCTION

Gaining a better understanding of the etiology and pathogenesis of psychiatric disease is currently a priority area of research (Campbell, 2010). Advances in human neuroimaging and genetics are giving insight into the cellular regions and processes involved. However, partly because studies in humans must deal with genetic, diagnostic and etiological heterogeneity as well as environmental (cultural, societ al) factors, and it is not possible to undertake molecular studies *in vivo*, progress remains slow (Burmeister et al., 2008). In order to address some of these concerns animal, primarily rodent, models targeting symptoms consistent with DSM-IV (APA, 2000) diagnoses of psychiatric disorder have been developed (Gould and Gottesman, 2006). Recently, with the establishment of zebrafish as a developmental genetic model with unparalleled utility for neural imaging, the potential of this genetically tractable vertebrate as a model in behavioral neuroscience has started to be realized (Levin and Cerutti, 2009; Norton and Bally-Cuif, 2010; Levin, 2011; Gerlai, 2012; Parker and Brennan, 2012).

In this paper, we review the current position with regards to the development and validation of zebrafish behavioral assays pertinent to human psychiatric disorder. We present an overview of neural pathways underlying key behaviors in rodents and the evidence for their conservation in fish. Finally we discuss prospects for the future: in particular, ways in which zebrafish can contribute to our understanding of cellular and molecular processes underlying psychiatric disease. Despite the numerous benefits of larval models and the progress that has been made in recent years (see Ahmad et al., 2012 for recent review), the utility of larvae to measure some of the subtle endophenotypes pertinent to vulnerability to psychiatric disorders may be limited. For example, although analysis of unconditioned or reflexive behaviors is clearly possible, it is unlikely that studying endophenotypes relative to cognitive or executive processes would be suitable owing to the immaturity of the larval neural systems. Thus here, we focus on adult behavioral phenotypes.

## THE BENEFITS OF DEVELOPING ZEBRAFISH MODELS OF BEHAVIORAL PHENOTYPES ASSOCIATED WITH PSYCHIATRIC DISEASE

There are a great many practical benefits of using zebrafish as a model organism, e.g., their small size and low housing costs, their transparent, rapidly developing, ex-utero embryos, and their unsurpassed genetic tractability. However, in the age of technological advances in *in vivo *methodologies, such as optogenetics, enabling modulation of cell physiology and activity at the single cell level, their utility may soon be even greater. In this section, we discuss the potential for using zebrafish for behavioral assays pertinent to psychiatric disease.

There are inherent difficulties in comparing behaviors observed in non-primate species – particularly those associated with psychiatric disease – with those seen in humans (Brown and Bowman, 2002). One key issue is the lack of an expanded telencephalon and prefrontal cortex (PFC), the primary areas controlling executive functions commonly disrupted in psychiatric disease. There did exist a central dogma in neuroscience that cognitive processes have evolved in concert with the expansion of the telencephalon and lamination of the cortex, and therefore, animals without this expansion cannot perform these behaviors. However, there is now increasing evidence that smaller-brained vertebrates, which lack expanded telencephali, are capable of cognitive processing and even complex decision-making. For example, non-mammalian brains that do not have a laminar structure, such as nucleated bird brains, (a) show complex cognition, (b) have similar neural and neurochemical systems (especially dopamine(DA) and (c) display executive functions like mammals which are controlled by homologous brain structures (nidopallium instead of PFC; Jarvis et al., 2005). Thus it seems that several species may have faced niche-specific selection pressures leading to evolution of comparable executive processes. This suggestion raises two questions regarding the use of zebrafish to explore molecular and cellular processes contributing to psychiatric disease: first, *do* fish show comparable behaviors? Second, if fish *can* perform executive tasks, have different regions or systems within their brains evolved to perform the tasks; or alternatively, have simple circuits (present in rudimentary form in common ancestors) evolved, albeit with topographical differences and different degrees of sophistication, to perform the same tasks, i.e., are the behavioral processes analogous or homologous? If the behaviors in fish, birds, and mammals share neurochemical pathways and show similar connectivity, it would suggest a common root and that the processes are homologous. In this event studies in fish, that do not model primate PFC executive function *per se,* can allow the extrapolation of common cell:cell interactions and physiological processes to give insight into molecules involved (in the human condition) despite differences in topography.

As the zebrafish is a uniquely tractable vertebrate genetic model species, assays of endophenotypes associated with psychiatric disease have been established and work to determine the neural pathways involved is underway. However, in the light of differences in structures, and possible differences in connectivity, there is a need for careful validation of the behavioral assays in zebrafish to establish their relevance to the human condition.

A good model of a human disease phenotype needs to demonstrate face-validity (the model *looks like* it is measuring the disease in question), construct validity (whether it *actually measures* what it claims to measure) and predictive power (Gould and Gottesman, 2006). As rodent models fulfill these criteria in many cases, zebrafish researchers have modified extant rodent assays, taking into account the specific behavioral system of the species. An example of this would be the novel tank test (Levin et al., 2007; Parker et al., 2012c). The novel tank test is an adaptation of the rodent open-field test of anxiety, developed with high face validity as it was designed with the zebrafish’s natural tendency to dive to the bottom of a new environment in mind. Construct validity has been demonstrated using anxiolytic drugs; buspirone; and diazepam reduce the amount of time spent in the bottom of the tank in a dose-dependent manner (Bencan et al., 2009). For other behaviors, rapid progress is being made and in many instances construct validity has been similarly established by the use of pharmacological manipulation and genetic loss-of-function lines. In this section, we will briefly describe some assays used in rodent models of psychiatric disease, and how these have been adapted for, and validated in, zebrafish. Although a number of different behavioral phenotypes can be linked to psychiatric disease, such as anxiety, in this review we will concentrate on disorders relating to executive functioning rather than affective state, which has been reviewed extensively elsewhere (e.g., see Maximino et al., 2010).

### BEHAVIORAL FLEXIBILITY

Deficits in cognitive or behavioral flexibility are commonly reported in patients with a variety of psychiatric diagnoses, such as somatoform disorders (anorexia nervosa, bulimia nervosa: Button et al., 2007; Cardona et al., 2011), bipolar disorder (Balleine and Dickinson, 1998), schizophrenia (Fernandez-Ruiz et al., 2001), substance abuse disorders (Collins et al., 2011) and obsessive compulsive disorder (OCD; Brembs, 2009). Behavioral flexibility is operationally defined as the ability to shift or adapt response strategy in the face of changing environmental contingencies (Ragozzino et al., 1999).

**FIGURE 1:**
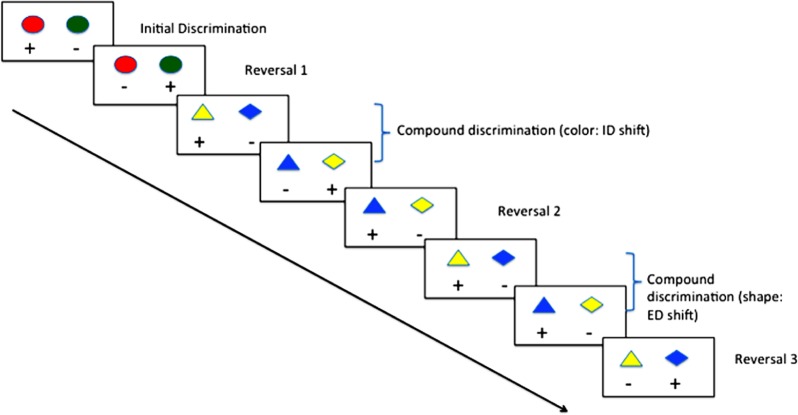
**Typical procedure for reversal learning with intra- (ID) and extra-dimensional (ED) set shifting using color and shape**.

In animal models, behavioral flexibility may be measured by serial reversal of contingencies in two-choice discriminations, or by intra- and extra-dimensional (ED) set-shift tasks (see **Figure 1**; Ragozzino et al., 1999). Convergence of evidence from pharmacological (Saus et al., 2010), lesion (Hoopes, 1999) and genetic knockout (White et al., 2008) studies suggest a high degree of construct validity when compared with human task performance on tests of behavioral flexibility. Typically, the assay involves the animal being trained to discriminate two stimuli (e.g., blue vs. green light), where in the first instance responses to the blue light are reinforced, and those to the green non-reinforced. Once the animal reaches a set criterion, the contingency is reversed, such that responses to the green light are now reinforced, and responses to the blue light non-reinforced. Subsequently, the colors can be changed (intra-dimensional [ID] shift) and reversed, or a third dimension can be introduced (e.g., shape; ED shift) and subsequently reversed. Many studies have demonstrated that rodents (Hoopes, 1999), primates (Doyle et al., 2006) and birds (Wilens et al., 2005) show a gradual improvement in their trials-to-criteria in this context.

Zebrafish are capable of reversal learning (Colwill et al., 2005), and recently they have been shown to follow a similar pattern of improvement over multiple reversals and ID shift as mammals. Parker et al. (2012a) trained adult zebrafish first on a simple color discrimination, once the fish had reached criterion (6 consecutive correct trials for 2 consecutive training sessions) the contingencies were reversed. Once the fish had reached criterion on the reversal condition, they were subjected to an ID shift, and subsequently contingency reversal. As has been shown in mammals, trials-to-criterion reduced during the course of the four training phases suggesting that the fish had formed an attentional-set, and could demonstrate flexibility in their learning with a changing environment. These findings were of particular interest, as they were the first to suggest that zebrafish were capable of behavioral flexibility in this context, complementing previous work in other fish species (e.g., goldfish; Woodward et al., 1971). Other, more ethological approaches to studying behavioral flexibility have also been used. Oliveira (2009), for example, observed that zebrafish adapt their social behavior dependent on outcomes of conflicts between conspecifics. These data raise the possibility of examining the cellular and molecular processes governing the operation of neural circuits involved in behavioral flexibility using zebrafish.

### ATTENTION

Attention can be described as *selective*, operationally defined as the ability to pick a target from an array of distracters (Desimone and Duncan, 1995), or *sustained*, operationally defined as the ability to detect the presence of a stimulus presented at various intervals over a prolonged period (Sarter et al., 2001). Deficits in sustained and selective attention are common features of a number of psychiatric disorders such as attention-deficit hyperactivity disorder (ADHD; Barkley, 1997), schizophrenia (Meshorer and Soreq, 2006), OCD (Kume et al., 2005), and substance abuse disorders (Tsutsui-Kimura et al., 2010). Both selective and sustained attention can be measured in a number of ways using animal models, and again, construct validity has been established using pharmacological, lesion and genetic models.

**FIGURE 2:**
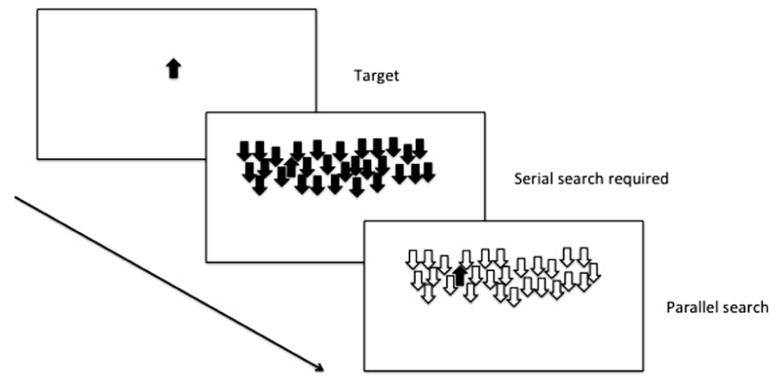
**Selective attention: parallel and serial search mechanisms are both used depending on the number of shared features with the distracters**.

In a widely used assay of selective attention (see **Figure 2**), an animal is initially trained to respond when a target cue is present. The target cue can then be presented among an array of distracter stimuli, and depending on the number of shared features with the target, the animal will either use serial search (i.e., scan every item in the array in order to locate the target) or parallel search (the entire scene is processed in parallel, and the target stimulus appears to “pop-out” of the array; Treisman and Gelade, 1980).

Selective attention can be inferred in zebrafish from their performance forming attentional sets in the reversal learning and ID-shift procedure described earlier (Parker et al., 2012a), but there is also promise to develop assays to examine more complex tasks examining serial and parallel processing in visual discrimination tasks. Zebrafish are effective at discriminating two stimuli in a variety of different conditions, under control of both aversive and appetitive contingencies (Levin and Chen, 2004; Colwill et al., 2005). In addition, further work that requires explicitly either top-down or bottom-up processing (Proulx and Serences, 2006) will provide an opportunity to explore the use of these attentional mechanisms in the zebrafish model.

**FIGURE 3:**
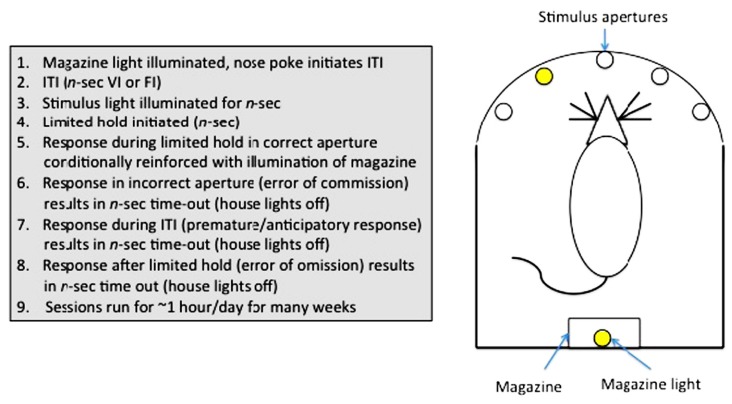
**Measurement of sustained attention and impulse control in rodents: The 5-choice serial reaction time task**.

Sustained attention can be assayed in humans using a continuous performance task (CPT; Rosvold et al., 1956). In animals, a variety of tests have been used (e.g., stop-signal task (Logan et al., 1997); go no-go task (Finn et al., 1999), but arguably the most useful has been the 5-choice serial reaction time task (5-CSRTT; Carli et al., 1983; Robbins, 2002), owing to the rich variety of parameters measurable in this assay. The 5-CSRTT (see **Figure 3**) tests the ability of animals to detect the presence of a briefly presented stimulus in one of five randomly ordered spatial locations following an inter-trial interval (ITI). Responses in the correct location during a limited time following the stimulus presentation (limited hold; LH) are conditionally reinforced with illumination of the magazine light, and subsequently food reinforcement, at the opposite end of the apparatus. Incorrect responses (errors of commission), anticipatory/premature responses during the ITI or failure to respond during the LH (errors of omission) are punished with a brief time-out. Subsequent trials are initiated with a nose-poke in the magazine. The 5-CSRTT can also be used to measure selective attention, as auditory or visual distracters can be added to the test environment during training or test sessions (Bari et al., 2008).

**FIGURE 4:**
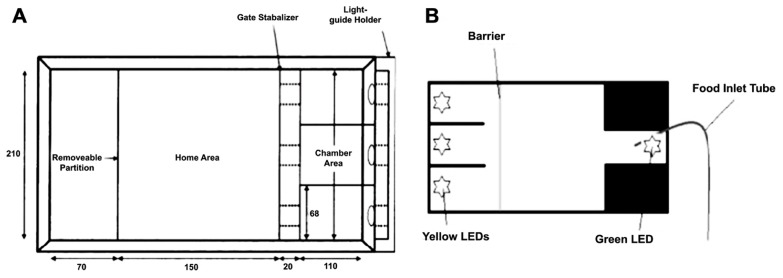
**Two different environments for assaying three-choice discriminations in zebrafish to measure sustained visual attention**. **(A)** adapted from Bilotta et al. (2005); **(B)** adapted from Parker et al. (2012b).

Sustained attention has been successfully demonstrated in zebrafish in two tasks (**Figure 4**). Initially, Bilotta et al. (2005) designed a task whereby fish were required to swim into one of three apertures. A light was illuminated in one of the chambers (see **Figure 4A**), and the barrier was lifted to allow the fish access to the choice area. The fish was reinforced for swimming into the correct chamber. Incorrect choices were punished by confining the fish to the incorrect chamber for 30-sec with no food. Fish were found to perform well on this task, quickly reaching 80% correct choices with repeated testing.

**FIGURE 5:**
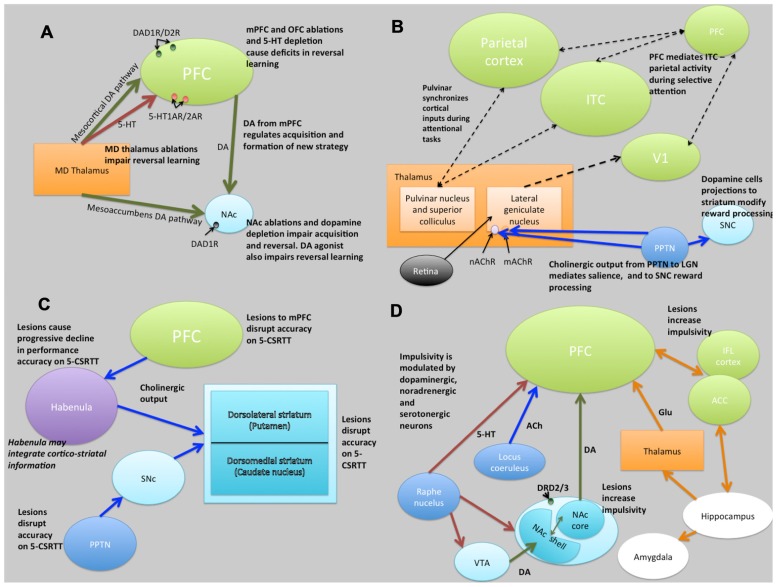
**Schematic illustrations of putative neural circuits of behavioral flexibility **(A)**, selective attention **(B)**, sustained attention **(C)**, and impulse control **(D)****. For detailed explanations see text. PFC, prefrontal cortices; MD Thalamus, medial dorsal thalamus; NAc, nucleus accumbens; DA, dopamine; 5-HT, 5-hydroxytryptamine (serotonin); ITC, infero-temporal cortex; V1, primary visual cortex (striate cortex); PPTN, pedunculopontine tegmental nucleus; SNc, substantia nigra pars compacta; nAChR, nicotinic acetylcholine receptor; mAChR, muscarinic acetylcholine receptor; LGN, lateral geniculate nucleus; PN, pulvinar nucleus; SC, superior colliculus; 5-CSRTT, 5-choice serial reaction time task; Ach, acetylcholine VTA, ventral tegmental area; ACC, anterior cingulate cortex; IFL cortex, infralimbic cortex; Glu, glutamate. Key: green lines, dopamine; orange lines, gluatamate; blue lines, acetylcholine; red lines, 5-HT; black dashed lines, multiple neurotransmitter pathways. Light green structures, cortical regions; orange structures, thalamic regions; light blue, basal ganglia/striatal (ventral and dorsal structures) regions; dark blue, brainstem structures; white, limbic structures; purple, other.

Another version of the task increased the complexity by: (1) requiring the zebrafish to maintain attention of the three stimulus apertures prior to making a response, and (2) by requiring the fish to return to the opposite end of the tank to receive their reinforcer (**Figure 5B**; Parker et al., 2012b). Both tasks have the ability to examine aspects of sustained attention, such as increasing attentional load by varying the duration or intensity of the stimuli, and hold great promise in terms of assessing attentional processes in zebrafish. In addition, it will be possible to adapt either of these tasks to include visual or auditory distracters to attempt to produce a parametric assessment of selective attention in zebrafish.

### IMPULSE CONTROL

Impulsive behaviors can be broadly divided into two categories: those resulting from deficits in the ability to withhold responding (impulsive action) and those that result from deficits in decision-making (impulsive choice). Impulsive action can be operationally defined in terms of anticipatory responding on assays such as the 5-CSRTT (Robbins, 2002), the go/no-go task (Finn et al., 1999), or the stop-signal reaction time task (Logan et al., 1997); impulsive choice is exemplified by the choice of a small, immediate reward over a delayed, more substantial reward (delay-discounting; Winstanley et al., 2006; Dalley et al., 2008; Diergaarde et al., 2008). Both categories of impulsive behavior are reported in a number of psychiatric conditions such as ADHD (Barkley, 1997), substance abuse disorder (Belin et al., 2008), pathological gambling (Alessi and Petry, 2003), schizophrenia (Winstanley et al., 2003) and OCD (Brembs, 2009). Animal (e.g., rodent) models of impulse control have been shown to have a good degree of construct validity, again using a combination of pharmacological, lesion and knockout studies.

Our lab has recently reported data pertaining to the performance of zebrafish in a three-choice version of the 5-CSRTT. Our version of the task is different from the rodent version, in that: 1) we use longer stimulus intervals (rodents = ~0.5-sec, fish = 5–10-sec), 2) we initiate the start of each trial by lifting the barrier to expose the stimulus apertures, while in rodents trials are initiated by nose-poking the magazine. Using this procedure, we demonstrated not only that zebrafish show similar rates of basal anticipatory responding on the task as rodents, but also that a low dose (0.025 mg/Kg i.p.) of amphetamine significantly reduced anticipatory responding during long ITI probe trials (low doses of psychostimulants reduce impulsivity in other animal models, and in humans; Robbins, 2002) whereas a saline injection had no effect (Parker et al., 2012b). In order to validate the procedure as a measure of impulse control, these findings need to be replicated with further pharmacological manipulations, and potentially with existing mutant lines. These preliminary findings are very encouraging, however, and we are in the process of automating the procedure to facilitate validation studies and future screening programs. Measures of impulsive choice, as measured in rodents by delayed reinforcement procedures, are currently lacking in zebrafish.

## NEURAL CIRCUITS MEDIATING BEHAVIORAL PHENOTYPES

In this section we outline what is currently known about the neural circuits that underlie the behaviors described above and their conservation in zebrafish.

### BEHAVIORAL FLEXIBILITY

Evidence from lesion studies suggests that regions of the PFC (medial pre-frontal cortex [mPFC]; orbitofrontal cortex [OFC]; lateral PFC), striatum (specifically the nucleus accumbens [NAc] and the dorsal-medial striatum [DMS]) and thalamic nuclei are involved with various aspects of behavioral flexibility (Ragozzino et al., 1999; Brown and Bowman, 2002; Ragozzino, 2007). Collectively, these data have suggested that reversal learning in mammals is mediated by cortical- striatal-thalamo-cortical loops (**Figure 5**). There is also much evidence to suggest that reversal learning is modulated by both DA and serotonin (5-HT). For example, impairments of reversal learning can be induced by DA depletion in the NAc (6-OHDA lesions of NAc: Taghzouti et al., 1985), by inhibition of DA reuptake with amphetamine (Ridley et al., 1981) or by 5-HT depletion in the PFC (Clarke et al., 2004). **Figure 5A** summarizes the putative circuits of behavioral flexibility in mammalian systems.

**FIGURE 6:**
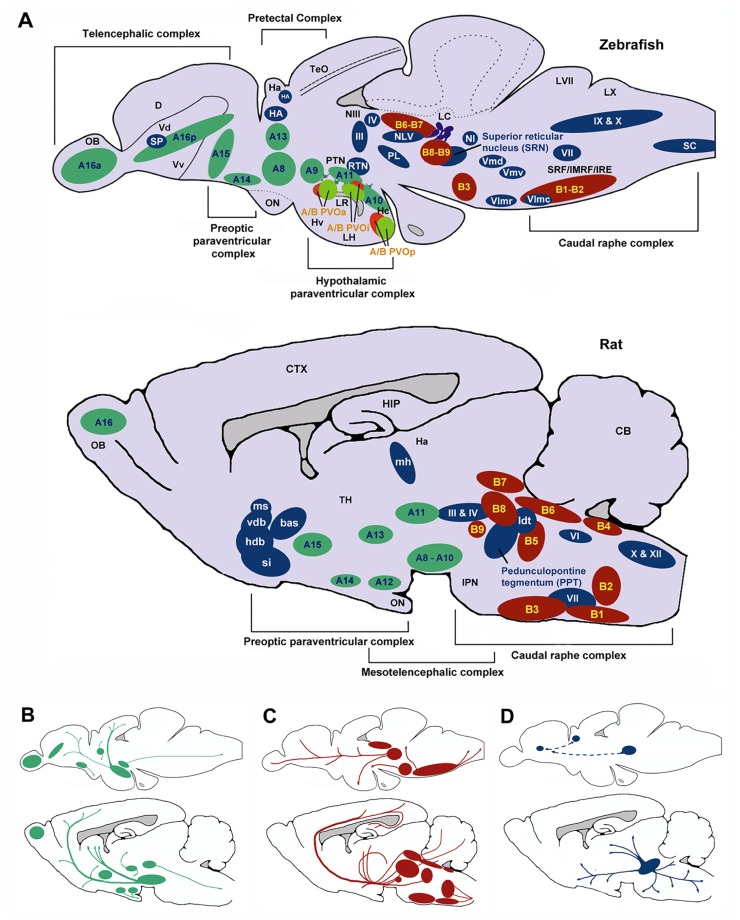
**Schematic sagittal view comparing dopaminergic (green), serotonergic (red), and cholinergic (blue) neuronal populations in zebrafish (upper) and rat (lower) brains**. Nomenclature for serotonergic and dopaminergic populations were based on their rostrocaudal distribution in the adult rat and zebrafish brain previously described (Schweitzer and Driever, 2009; Panula et al., 2010) allowing comparison between cell body distribution in adult brains. **(A)** Cell body distribution (adapted from Manger et al., 2002; Butcher and Woolf, 2003; Mueller et al., 2004; Schweitzer and Driever, 2009; Panula et al., 2010). Corresponding **(A,B)** nomenclature for dopaminergic and Serotonergic cell bodies derived from Panula et al. (2010) and Schweitzer and Driever (2009). Dopaminergic populations (zebrafish): A8: parvocellular preoptic nucleus, posterior part (PPp); A9: periventricular nucleus of posterior tuberculum (TPp); A10: periventricular hypothalamus and posterior tuberculum (PTN); A11: posterior tuberculum (PTN); A13: anterior, intermediate, ventrolateral, and ventromedial thalami nuclei (A, I, VM, and VL); A14: parvocellular preoptic nucleus, anterior part (PPa); A15: parvocellular preoptic nucleus, anterior part (PPa); A16p: ventral telencephalic nuclei (Vv, Vd and Vs); A16a: olfactory bulb (Ob). Dopaminergic populations (rat): A8: midbrain reticular formation (MRF); A9: ventrolateral midbrain (VLM); A10: ventral midbrain tegmentum (VTAe); A11: posterior periventricular nucleus and intermediate periventricular nucleus (PVNP and PVNI); A12: hypothalamic arcuate nucleus (ARH); A13: mammillothalamic tract (MTT); A14: anteroventral periventricular nucleus (AVPV); A15: Anteroventral periventricular nucleus (AVPV); A16: olfactory bulb (OB). Serotonergic populations (zebrafish): B1-B2: caudal raphe complex; B3-B9: rostral raphe complex; BPVOa: paraventricularorgan, anterior part (PVOa); BPVOi: paraventricular organ, intermediate part (PVOi); BPVOp paraventricularorgan posterior part (PVOp). Serotonergic populations (Rodent): B1: raphe pallidus (RPa); B2: raphe obscurus (NRO); B3: raphe magnus (NRM); B4: vestibular nucleus (VN); B5: pontine raphe nucleus (PRN); B6: medial longitudinal fasciculus (MLF); B7: dorsal raphe nucleus (DRN); B8: median raphe nucleus (MRN); B9: median raphe nucleus (MRN). Cholinergic populations (zebrafish): IX: glossopharyngeal nerve motor nucleus; X: vagal nerve motor nucleus; SC: spinal cord motoneurons; NI: nucleus isthmi; HA: Habenula; NLV: nucleus lateralis valvulae; III: oculomotor nerve nucleus; IV: trochlear nerve motor nucleus; RTN: rostral tegmental nucleus; PL: perimeniscal nucleus; Vmd: dorsal trigeminal nerve motor nucleus; Vmv: ventral trigeminal nerve motor nucleus; VII: facial nerve motor nucleus; VImr: rostral abducens nerve motor nucleus; VImc: caudal abducens nerve motor nucleus. Cholinergic populations (rat): ldt: lateral dorsal tegmental nucleus; si: substantia innominate; hdb: horizontal diagonal band nucleus; vdb: vertical diagonal band nucleus; bas: nucleus basalis; ms: medial septal nucleus (MS); md: medial habenula (MH); III: oculomotor nucleus (IIIn); IV: trochlear nucleus (IVn); VII: facial nucleus (VIIn); X: dorsal motor vagus nucleus (Xn); XII: hypoglossal nucleus (Xn). SP: Subpalium (Sp). **(B) **Schematic drawing illustrating the location of dopaminergic projections in adult zebrafish and rat brains (sagittal view; adapted from Schweitzer and Driever, 2009). **(C) **Schematic drawing illustrating the location of serotoninergic projections in adult zebrafish (adapted from Gaspar and Lillesaar, 2012) and rat brains (adapted from Di Giovanni et al., 2008; sagittal view). **(D)** Schematic drawing illustrating the location of cholinergic neuron projections from PPT in adult rats (adapted from (Manger et al., 2002)) and predicted projections from zebrafish SRN to subpallium and habenula. D, dorsal telencephalic area; Dc, caudal dorsal telencephalic area; CTX, cerebral cortex CB, cerebellum; Ha, habenula; Hc, caudal zone of periventricular hypothalamus; HIP, hippocampus; Hv, ventral zone of periventricular hypothalamus; IMRF, intermediate reticular formation; IPN, interpeduncular nucleus; IRF, inferior reticular formation; LC, locus coeruleus; LH, lateral hypothalamic nucleus; LR, lateral recesses of the diencephalic ventricle; LVII, facial lobe; LX, vagal lobe; NIII, oculomotor nucleus; OB, olfactory bulb; ON, optic nerve; OBN, olfactory bulboptic nerve; PTN, posterior tuberculum; PVO, paraventricular organ, anterior part; PVOi, paraventricular organ, intermediate part; BPVOp, paraventricular organ posterior part; SRF, superior reticular formation; Vd, dorsal telencephalic area; Vv, ventral telencephalic area; TeO, optic tectum; TH, thamalus.

The topography of the zebrafish brain differs from the mammalian brain, but homologues for the different brain regions have been identified. For example, both the mammalian and the zebrafish thalamic nuclei are located in the diencephalon (**Figure 6**), and homologues of midbrain regions such as the VTA (posterior tuberal nucleus; PTN) and NAc (ventral [Vv] and dorsal [Vd] telencephalic nuclei; Rink and Wullimann, 2002; Panula et al., 2010) have been determined. Recently, Mueller et al. (2011) have also identified the central region of the dorsal pallium (area Dc in **Figure 6**) as a potential homologue of the isocortex, that in mammals encompasses the above-mentioned cortical regions. There are a number of neurochemical pathways relevant to behavioral flexibility that have a good degree of homology. For example, the ascending midbrain DA pathways have been well characterized using tyrosine hydroxylase (TH) immunohistochemistry (Rink and Wullimann, 2001, 2002; Filippi et al., 2010), with a number of putative functional homologues being identified owing to their neuronal connections and projections (**Figure 6**). Much of the evidence pertaining to the cholinergic, DAergic and 5-HTergic neural clusters in the zebrafish brain has been generated from extensive immuno-staining of relevant cell bodies. There is little evidence of how the systems functionally interact, and no direct evidence for a homologous circuit for the cortico-striatal-thalamo-cortical loops. Specifically, whether or not the neural clusters are reciprocally connected remains to be seen.

### SELECTIVE ATTENTION

**Figure 5B** summarizes the putative neural circuits of selective attention in mammalian systems. The cholinergic system plays a central role in selective attention (Robbins, 1997). Cholinergic neurons project widely to such basal forebrain structures as the habenula (Claudio Cuello et al., 1978) and the striatum (Woolf and Butcher, 1986), releasing ACh at various synaptic terminals across all layers of the cortex via activation of cholinergic receptor sub-types (Sarter and Bruno, 1997). ACh binds to two distinct categories of cholinergic receptors: G-protein coupled metabotropic muscarinic receptors (mAChR) and ligand-gated ionotrophic nicotinic receptors (nAChR), both of which are implicated in attention (Noudoost and Moore, 2011). In addition, the pedunculopontine tegmental nucleus (PPTN) may play a dual role in selective attention pertaining to the processing of saliency and reward-cues. Specifically, cholinergic outputs from the PPTN synapsing on lateral geniculate nucleus (LGN) cells expressing nAChRs and mAChRs regulate saliency, and those synapsing on substantia nigra pars compacta (SNc) DAergic cells regulate reward processing (Kobayashi and Isa, 2002).

The role of the DA system in attention is also well established (Nieoullon, 2002). For example, people with Parkinson’s disease (characterized by a loss of nigrostriatal DA neurons; Owen et al., 1993) and ADHD (characterized by reductions in PFC DA; Barkley, 1997) both show deficits in selective attention, as do people with schizophrenia (characterized by increases in D2-like receptors in the striatum; Seeman et al., 1993). Although not universally accepted, there is some evidence that ACh modulation may relate more to aspects of saliency (Asadollahi et al., 2010; Knudsen, 2011), and DA more to motivated search. As such, it may be that the roles of ACh and DA are, respectively, related to bottom-up and top-down processing in a dissociated, or at least partially dissociated manner (Noudoost and Moore, 2011).

In terms of homologous brain regions involved in cholinergic signalling, the cortical regions thought to be involved with selective attention are relatively well conserved in zebrafish, although the topography is, again, somewhat different (**Figure 6**). Thus, the cholinergic projections from the PPTN to the brainstem, habenula and thalamic (e.g., LGN) regions in rodents are mirrored by projections from the superior reticular nucleus (SRN) to the brainstem, habenula and subpallium in fish. As discussed above, the neurotransmitter systems thought to modulate selective attention (DA, ACh) are well conserved in zebrafish (see **Figure 6**). For example, the mAChRs and nAChRs have been identified in zebrafish and their binding characteristics and expression patterns described (Zirger et al., 2003). Similarly, the DA system has been characterized and the homologues of key components of the reward pathway identified as discussed above (Filippi et al., 2010). In the light of this, neuromodulatory influences may be conserved between the species.

### SUSTAINED ATTENTION

The frontal cortices (frontal executive system) are thought to mediate sustained attention (Barkley, 1997). Indeed, some theories of ADHD (which is characterized, in part, by difficulties in sustained attention) suggest that the symptoms may be caused by delayed cortical maturation (Loo and Barkley, 2005). Although the circuits underlying sustained attention are less clearly defined, lesions studies have indicated brain regions involved (see **Figure 5C**). Notably, the PPTN has outputs via the SNc to striatum and to PFC suggesting a role of the nigrostriatal DA pathway in some aspects of sustained attention, perhaps relating to motor control during tasks requiring sustained attention. In addition to the frontal cortices, the striatum appears to be crucially linked to aspects of sustained attention (Barkley, 1997).

The cholinergic system, including cholinergic neuronal projections and cholinergic receptors (mAChR and nAChR), plays a crucial role in sustained attention. Cortical ACh is released during tasks requiring sustained attention (Arnold et al., 2002), and as the task increases in difficulty, the release of ACh increases (Himmelheber et al., 2000). The DAergic system has also been strongly linked to sustained attention, in particular relating to the dissociable roles of D1 and D2 receptors in the mPFC. **Figure 5C** summarizes the putative neural circuits of sustained attention.

As mentioned above, homologous regions exist and the midbrain DAergic system has been well characterized in zebrafish. In addition to this, the cholinergic system in zebrafish has been characterized with choline acetyltransferase (ChAT) immunohistochemistry (Mueller et al., 2004; **Figure 6**). Cholinergic cell bodies are found in the caudal raphe complex of both mammals and zebrafish (**Figure 6**). Of particular note here, zebrafish have an ascending SRN cholinergic system which is a putative homologue for the mammalian PPTN, lesions of which are known to impair sustained attention in rats (Kozak et al., 2005).

### IMPULSE CONTROL

Impulse control and motivational circuits are inextricably linked. Motivational circuits have been extensively characterized (Dalley et al., 2008; **Figure 5D**). As is the case with behavioral flexibility, lesions to the medial dorsal, but not anterior, thalamic nucleus, cause increases in premature responding (Chudasama and Muir, 2001). The data strongly suggest that impulsivity, at least that form of impulsivity measured by the 5-CSRTT, is mediated by DAergic and 5-HTergic cortico-striatal-thalamo-cortical loops (Robbins, 2002). Clearly this suggests a role for catecholaminergic systems in impulsivity, and indeed high doses of systemic amphetamine administration increases premature responding, an effect that is reversed by 6-OHDA lesions of the NAc (Cole and Robbins, 1989). In addition, systemic atomoxetine, a noradrenergic reuptake inhibitor, reduces impulsivity (Robinson et al., 2008). Finally, rats characterized as “high-impulsive” on the 5-CSRTT have reduced DA D2/3 receptors in the NAc (Dalley et al., 2007). **Figure 5D** summarizes the putative circuits of impulse control in mammalian systems.

The neural circuits currently hypothesized to modulate impulse control (e.g., ascending midbrain DA pathways and raphe complex 5-HT pathways; **Figure 6B**) are present in zebrafish, or at least, putative functional homologues exist (**Figure 6**). For example, the caudal raphe complex is well conserved between species, as are the 5-HT projections from this region to prefrontal regions in rats and dorsal pallial regions in the zebrafish. In addition, homologues for the VTA (PTN), NAc (Vv and Vd; Rink and Wullimann, 2002; Panula et al., 2010) and area Dc (Mueller et al., 2011) have been identified and DA projections in these regions are similar. In the zebrafish brain DAergic and 5-HTergic projections from the pallium to the thalamic nuclei, in addition to DAergic projections from the telencephalic nuclei to the pallium (see **Figure 6**), suggest similar patterns of connectivity to mammalian brains.

## FUTURE PROSPECTS: HOW CAN ZEBRAFISH CONTRIBUTE TO THE UNDERSTANDING OF EXECUTIVE FUNCTION AND THE ETIOLOGY OF PSYCHIATRIC DISEASE?

To date researchers interested in using zebrafish to study executive function have focused on the development of appropriate behavioral paradigms. Now that many assays have been established zebrafish are well placed to address key questions relating to the control of these behaviors, particularly in the areas of impulse control and attention, and the etiology of disease: (1) Which neural circuits are involved and how do they develop? (2) What are the genetic factors that influence impulse control and attention? (3) What are the cell biological processes by which genetic factors act to influence these behaviors and contribute to behavioral disease? The primary advantages zebrafish have in the search for knowledge in these areas are their transparency, which facilitates *in vivo* analysis of the development and functioning of neural circuits controlling behavior, and the ability to perform large scale pharmacological and genetic screens.

### DISSECTION OF THE NEURAL CONTROL OF BEHAVIOR

The information regarding brain regions and pathways involved in the rodent behaviors described above were in large part obtained using lesion studies and pharmacological manipulations. These approaches are relatively crude as manipulations often affect surrounding cell types and processes or have slow reversibility (Mei and Zhang, 2012). In contrast, the use of optogenetic techniques that combine the use of light-controlled reporters and manipulators of neuronal activity with genetic targeting, allows more precise dissection of the neural control of behavior. A number of different optogenetic constructs for manipulating neuronal activity are available for use in fish as in other species. For example, channelrhodopsin cation channels can activate neurons by depolarising the membrane potential upon activation by light, whereas halorhodopsin and bateriorhodopsin channels act as light sensitive chloride and proton pumps capable of hyperpolarizing the membrane thus inhibiting action potentials. Although utilized in many model organisms, zebrafish are particularly well suited to the application of optogenetic techniques. In transparent larval and, to lesser extent, adult *casper* (White et al., 2008) forms, we have the ability to drive expression in specific cell types using GAL4:UAS constructs (Davison et al., 2007; Scott et al., 2007; Asakawa and Kawakami, 2008; Asakawa et al., 2008) or transposon technologies (Petzold et al., 2009). This provides an almost entirely non-invasive method for visualizing and modulating neuronal activity at even the single cell level and examining the effect on behavior. By comparison, in other animal systems, such as murine or primate models, holes must be drilled into the skull and fiber optic cables inserted to have access to the brain and control behavior *in vivo* with light optics (Cao et al., 2010). As the application of optogenetic techniques to address development and functioning of neural circuits in zebrafish have been extensively discussed elsewhere (Schoonheim et al., 2010; Del Bene and Wyart, 2012; Portugues et al., 2012; Umeda et al., 2012), in the section below we summarize more recent advances in technologies to visualize developing neuronal circuits.

Recent technological advances based on the use of genetically encoded GFP variants allow individual projections (*Brainbow*; Pan et al., 2011) and synaptic contacts GFP reconstituted across synaptic partners (GRASP) to be resolved, and activity within neuronal circuits (GCaMP) to be followed using fluorescence microscopy. *Brainbow* relies on combinatorial expression of several fluorophores (XFPs) to label individual neurons and their projections. Using Cre-lox recombination technology under the control of neuron specific promoters, each individual neuron expresses a random combination of each of up to four different XFPs to generate a specific fluorescence signal. As each neuron expresses the four XFPs at different levels, up to 100 different spectra can be obtained. Using this technology it is possible to trace the neuronal projections and formation of neuronal circuits in developing embryos and larvae. Pan et al. (2011) used this approach to map the zebrafish trigeminal projections.

“GFP reconstituted across synaptic partners” technology developed in *c. elegans* (Feinberg et al., 2008) can be used to trace the formation of synaptic contacts at high resolution *in vivo*. GRASP involves the expression of complementary fragments of GFP tethered to extracellular domains of transmembrane carrier proteins on pre- and post-synaptic membranes. The individual fragments of GFP are not fluorescent, but the formation of a synapse brings the two fragments into close proximity allowing reconstitution of the fluorescent molecule. Although application of this technique to zebrafish circuits has yet to be published, it has been used to map neuronal connections in *c. elegans*, *Drosophila* and mice (Feinberg et al., 2008; Arenkiel and Ehlers, 2009; Kim et al., 2011) and has the potential to trace synapse formation *in vivo* in wild-type and behaviorally mutant zebrafish.

In addition to tracing neuronal projections and synapse formation, fluorescence, including GFP technologies, have been used to monitor activity within neuronal populations in living behaving larval zebrafish. For example, the group of Rainer Friedrich (Li et al., 2005) has used whole brain calcium imaging to track activity within neuronal olfactory circuits as larval fish respond to changes in olfactory cues. Herwig Baier’s group (Del Bene et al., 2010) used GCaMP reporter constructs under the control of neuron specific promoters to identify neural circuits involved in processing visual information. Using a similar approach, Koichi Kawakami’s group (Muto and Kawakami, 2011; Muto et al., 2011, 2013) have identified circuits involved in spontaneous motor behavior and perception in embryonic zebrafish. When coupled with optogenetic approaches to manipulate activity in specific cells, these techniques provide a powerful means of dissecting neuronal circuits controlling behavior. Another particularly elegant larval assay that could be used to address the neurobiology of selective attention was recently described. Bianco et al. (2011) tracked the eye convergence and body position in 7dpf larvae, partially restrained in agarose, in response to different sized virtual stimuli. They found that the larvae tracked small moving spots, adopted a J-bend of their tail (the body shape that precedes prey capture; McElligott and O’Malley, 2005) and showed eye convergence on the target. This initial evidence of oculomotor processing of prey provides some evidence of a basic form of saliency-based selective attention, which may hold great promise for the development of tests of executive function in zebrafish in the future. As discussed by Bianco et al. (2011), by combining the assay with functional imaging of genetically encoded calcium indicators (Higashijima et al., 2003), techniques to manipulate circuits (Douglass et al., 2008; Janovjak et al., 2010; Schoonheim et al., 2010), and targeted laser-ablations (McLean et al., 2007; Satou et al., 2009; Burgess et al., 2010) it will be possible to identify the neuronal circuits controlling this, and similar, behavior.

Although the majority of these fluorescence techniques have to date only been applied to larval fish, advances in multiphoton confocal technology and computer processing raises the possibility of performing similar studies in juvenile and adult fish in the future. These imaging approaches coupled with the large-scale mutagenesis analysis possible in zebrafish have unprecedented potential to extend understanding of the cellular and molecular bases of behavior

### FORWARD GENETIC SCREENING

The ability to perform forward genetic screens for behavioral phenotypes also has great potential to advance understanding of the neurobiology of behavior. Forward genetic mutagenesis screens in zebrafish have been widely used to identify mutant alleles affecting developmental phenotypes. The classic three-generation mutagenesis screen for recessive alleles looks for families in which 25% of the F3 offspring show a given phenotype. This approach works well for recessive (or dominant) alleles of major effect but has, thus far, proved less effective for complex behavioral phenotypes likely to be governed by multi-allelic variations, each of minor effect and variable penetrance. Nonetheless, forward genetic screens for behavioral phenotypes have been undertaken. Darland and Dowling (2001) and Ninkovic et al. (2006) performed screens for cocaine and amphetamine-induced place preference, respectively. Both isolated lines of fish with differential drug seeking behavior, but neither have successfully isolated the causal mutations, possibly due to difficulties in unambiguously identifying the mutant carrier; the performance of control individuals often falls within the range of affected individuals and vice versa (Jain et al., 2011) making linkage analysis difficult.

Population based breeding and selection, or GFP insertion techniques can be used to address this problem. For example, Jain et al. (2011) used a “phenotyping by segregation” approach, based on commonly used breeding and selection strategies, to map the hypersensitive zebrafish *houdini* mutant. This strategy is attractive as it allows for fine mapping of subtle phenotypes that may have variable penetrance in the general population. An alternative approach taken by Petzold et al. (2009) used fluorescently tagged gene breaking transposons to mutagenize zebrafish. These transposons permit visual sorting of carriers from non-carriers (fluorescent vs. non-fluorescent larvae) and have the advantage of allowing rapid cloning of the mutagenized gene. Petzold et al. (2009) successfully used this insertional mutagenesis approach to identify two genes involved in the behavioral response of larval fish to nicotine.

Application of such breeding and selection-based and insertional mutagenesis screening approaches to the adult behavioral assays outlined above may lead to the identification of novel genes contributing to complex behavioral phenotypes. Such studies will make a valuable contribution to complement genome wide association studies (Sullivan, 2010) and analyses of copy number variants (Cook and Scherer, 2008) aimed at understanding the genetics of psychiatric disease.

### IDENTIFYING CELL BIOLOGICAL PROCESSES AFFECTING COMPLEX BEHAVIORS

Genome-wide association studies in humans are excellent tools for identifying genetic variations associated with psychiatric disorder phenotypes (Mitchell and Porteous, 2009; Ersland et al., 2012). In some instances these studies are even being able to identify endophenotypes associated with multiple psychiatric disorders (Hall and Smoller, 2010; Consortium, 2013). However, while being able to identify genetic markers associated with a particular disorder, they are not able to establish which variations are of causal effect. The ability in zebrafish to generate targeted knockouts of candidate genes using TALEN technology (Sander et al., 2011) offers a cost effective and convenient means of investigating which of the candidate alleles identified in human GWAS studies are causally linked to behaviors. Further, once an allele of effect is identified, zebrafish provide an ideal model system in which to investigate the neurodevelopmental and cellular processes affected.

## CONCLUSION

In this review, we have discussed the potential for using zebrafish to uncover some of the molecular and cellular processes related to psychiatric disorder, in particular relating to disorders of executive function. These are exciting times for zebrafish researchers. New assays of subtle behavioral phenotypes are fast being developed and replicated in different laboratories, and validation of these phenotypes is underway and progressing well. Given the huge repertoire of genetic tools available and the ever-expanding mutant resource, zebrafish will soon become one of the leading animal models in behavioral neuroscience. Even in the event that there are significant differences in anatomy or connectivity, many of the behaviors we have discussed are extensions of evolutionarily ancient reward and impulse control processes that appear to have conserved neurochemical pathways. In this regard, understanding the molecular mechanisms regulating these processes in fish will still give insight into regulation in mammals. As a final thought, there still remains much debate over how best to describe even simple neural circuits, and at this point no vertebrate system, regardless of technological advances, can come close to dealing with this issue (Yuste, 2008). Even in the age of the Human Connectome Project, the complexities of characterizing functional neural circuits should not be underestimated (Koch, 2012).

## Conflict of Interest Statement

The authors declare that the research was conducted in the absence of any commercial or financial relationships that could be construed as a potential conflict of interest.
